# Identification of a Chitooligosaccharide Mechanism against Bacterial Leaf Blight on Rice by In Vitro and In Silico Studies

**DOI:** 10.3390/ijms22157990

**Published:** 2021-07-27

**Authors:** Supatcharee Siriwong, Wannaporn Thepbandit, Nguyen Huy Hoang, Narendra Kumar Papathoti, Karsidete Teeranitayatarn, Tippawun Saardngen, Kanjana Thumanu, Sundaresan Bhavaniramya, Vaseeharan Baskaralingam, Toan Le Thanh, Piyaporn Phansak, Natthiya Buensanteai

**Affiliations:** 1 School of Crop Production Technology, Institute of Agricultural Technology, Suranaree University of Technology, Nakhon Ratchasima 30000, Thailand; supatcharee@slri.or.th (S.S.); w.thepbandit@gmail.com (W.T.); huyhoangqct@gmail.com (N.H.H.); narendrakumar.papathoti@gmail.com (N.K.P.); 2 Synchrotron Light Research Institute (Public Organization), Nakhon Ratchasima 30000, Thailand; kthumanu@gmail.com; 3 Green Innovative Biotechnology Co., Ltd., Nonthaburi 11110, Thailand; gibardc@gib.co.th (K.T.); tippawun@gib.co.th (T.S.); 4 Crustacean Molecular Biology and Genomics Lab, Alagappa University, Karaikudi, Tamilnadu 630004, India; sbhavaniramya@gmail.com (S.B.); vaseeharanb@gmail.com (V.B.); 5 Department of Plant Protection, Can Tho University, Can Tho City 900000, Vietnam; lttoan@ctu.edu.vn; 6 Division of Biology, Faculty of Science, Nakhon Phanom University, Muang Nakhon Phanom 48000, Thailand; pphansak@npu.ac.th

**Keywords:** bacterial leaf blight, biochemical alterations, chitooligosaccharides, synchrotron FTIR, drug target

## Abstract

This study focuses on a commercial plant elicitor based on chitooligosaccharides (BIG^®^), which aids in rice plant growth and disease resistance to bacterial leaf blight (BLB). When the pathogen (*Xoo*) vigorously attacks rice that has suffered yield losses, it can cause damage in up to 20% of the plant. Furthermore, *Xoo* is a seed-borne pathogen that can survive in rice seeds for an extended period. In this study, when rice seeds were soaked and sprayed with BIG^®^, there was a significant increase in shoot and root length, as well as plant biomass. Furthermore, BIG^®^-treated rice plants showed a significant reduction in BLB severity of more than 33%. Synchrotron radiation-based Fourier transform infrared (SR-FTIR) analysis was used to characterize BIG^®^’s mechanism in the chemical structure of rice leaves. The SR-FTIR results at 1650, 1735, and 1114 cm^−1^ indicated changes in biochemical components such as pectins, lignins, proteins, and celluloses. These findings demonstrated that commercial BIG^®^ not only increased rice growth but also induced resistance to BLB. The drug’s target enzyme, *Xoo* 1075 from *Xanthomonas oryzae* (PDB ID: 5CY8), was analyzed for its interactions with polymer ingredients, specifically chitooligosaccharides, to gain molecular insights down to the atomic level. The results are intriguing, with a strong binding of the chitooligosaccharide polymer with the drug target, revealing 10 hydrogen bonds between the protein and polymer. Overall, the computational analysis supported the experimentally demonstrated strong binding of chitooligosaccharides to the drug target.

## 1. Introduction

Bacterial leaf blight (BLB), caused by *Xanthomonas oryzae pv. oryzae (Xoo)*, is a significant rice disease. The disease is widespread in Thailand and other Asian countries. When *Xoo* vigorously attacks rice that has suffered yield losses, its damage can reach more than 20%. Furthermore, *Xoo* is a seed-borne pathogen that can survive for a long time in rice seeds. Pathogenic bacteria can first appear in the middle of the plant’s top foliage and then spread throughout the other rice plants [1]. Chemical control has been used in the past, but its effectiveness is low during the rainy season when there is high environmental pollution [2]. In recent years, high-level resistance elicitors have been tested to reduce crop disease damage based on the principles of induced systemic resistance (ISR), acquired systemic resistance (SAR), and induced resistance (IR) [3]. Plants have innate immunity that allows them to resist infection by means of a wide range of phytopathogens. Once the resistance mechanism is established within the plant, the protective components are strengthened, resulting in resistance to future phytopathogenic infections [4,5].

Natural and synthetic compounds can be used as elicitors. Chitooligosaccharides (COS) are natural biopolymers formed by the deacetylation of chitin in crustacean exoskeletons. Cell lysis, disruption of the cytoplasmic membrane barrier, and inhibition of RNA and protein synthesis, as well as chelation of the trace metal cations that may be required for the microorganism’s growth and survival are all proposed mechanisms of bacterial inhibition by chitosan. The most widely accepted mechanism is the interaction of positively charged chitosan with negatively charged residues on the cell surface of many fungi and bacteria, which results in extensive cell surface alterations, as well as changes in cell permeability. Furthermore, several studies [5,6,7] have shown that chitosan can induce resistance against bacterial pathogens in plants exposed to it. Although the use of natural plant elicitors to control crop diseases is widespread, Thailand needs to assess the effectiveness of BLB disease control. Although previous research has shown that COS can prime a defense mechanism against various pathogens in plants, no commercial formulation of COS has been developed in Thailand. Green Innovative Biotechnology Co., Ltd. (Bangkok, Thailand) recently developed a COS formulation known as BIG^®^ (with COS as its active ingredient).

**Figure 1:**
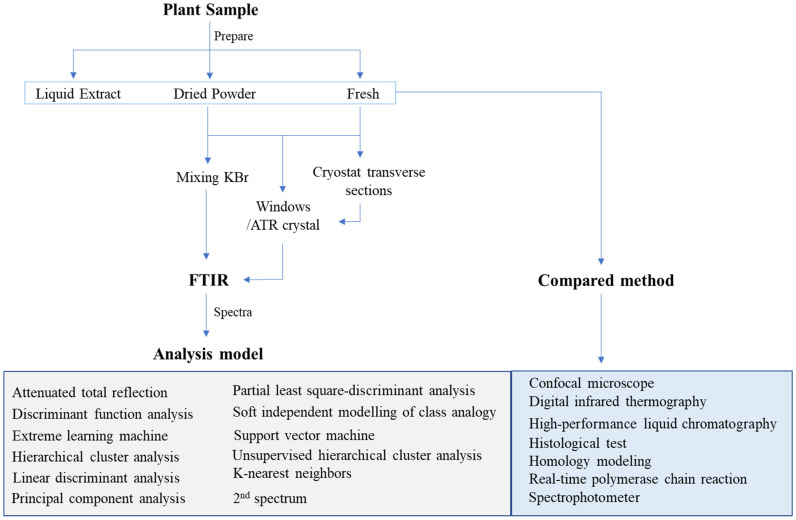
The research schematic for applying FTIR in plant disease.

In synchrotron (SR) FTIR microspectroscopy, a biochemical analysis technique, the mid-IR region between 400 and 4000 cm^−1^ is observed in the chemical component of a biological sample, with the vibrations at various frequencies from different types of bonds providing a highly specific profile. The IR spectrum of CH3 and CH2 at 2800–3000 cm^−1^ is defined as asymmetric and symmetric. The absorbance peak at 1739 cm^−1^ is caused by the C=O ester of pectin. The stretching vibration of the C=C aromatic skeleton from lignin results in a region of 1515 cm^−1^. The region induced by functional groups of C-O-C glycosides, primarily hemicelluloses or polysaccharides, is between 1200 and 900 cm^−1^ [8]. However, this histochemical analysis does not explain the distinction between susceptible and resistant pathogen strains. Plant pathogens are responsible for biomolecules in plant tissues, which can be used to explain disease resistance mechanisms in plants. Disease defense is associated with support of the plant cell wall, which is the most important barrier in the plant [9]. The mechanism of resistance of mutant wheat to disease has been compared before and after fungal pathogen inoculation. The defense mechanism was manifested as changes in biochemical components. After inoculation, the distribution of proteins and lignins in the tissue increased to protect fungal penetration into the cell by increasing cell wall integrity and activating pathogenesis-related (PR) proteins [10]. Figure 1 depicts the research schematic for using FTIR in the study of plant disease.

Molecule interaction fields (MIFs) were first used in computational studies in the late 1970s to describe the three-dimensional interactions that occur between a molecule and its surroundings. Despite significant differences in their underlying chemical structures, molecules that elicit similar interactions [11] produce similar MIFs. This paradigm enables a variety of important scientific tasks, such as bioisosteric replacement and molecular similarity assessments, as well as virtual screening, quantitative structure–activity relationships, and metabolic liability predictions [5]. However, the precise mechanism of action of chitosan’s inhibitory activity against pathogenic bacteria in plants remains unknown. In this study, we focus on the efficacy of the BIG^®^ natural plant elicitor in the control of BLB disease and investigate biochemical changes in rice by monitoring the development of induced resistance markers induced by the BIG^®^ natural plant elicitor against bacterial leaf blight disease using SR-FTIR microspectroscopy combined with multivariate data analysis. MIF-based in silico methodologies were applied to chitooligosaccharides in the peptide deformylase enzyme of the pathogen (*Xoo*) using both ligand- and structure-based molecular designs.

## 2. Results

### 2.1. Efficacy of BIG^®^ on Rice Growth

**Figure 2:**
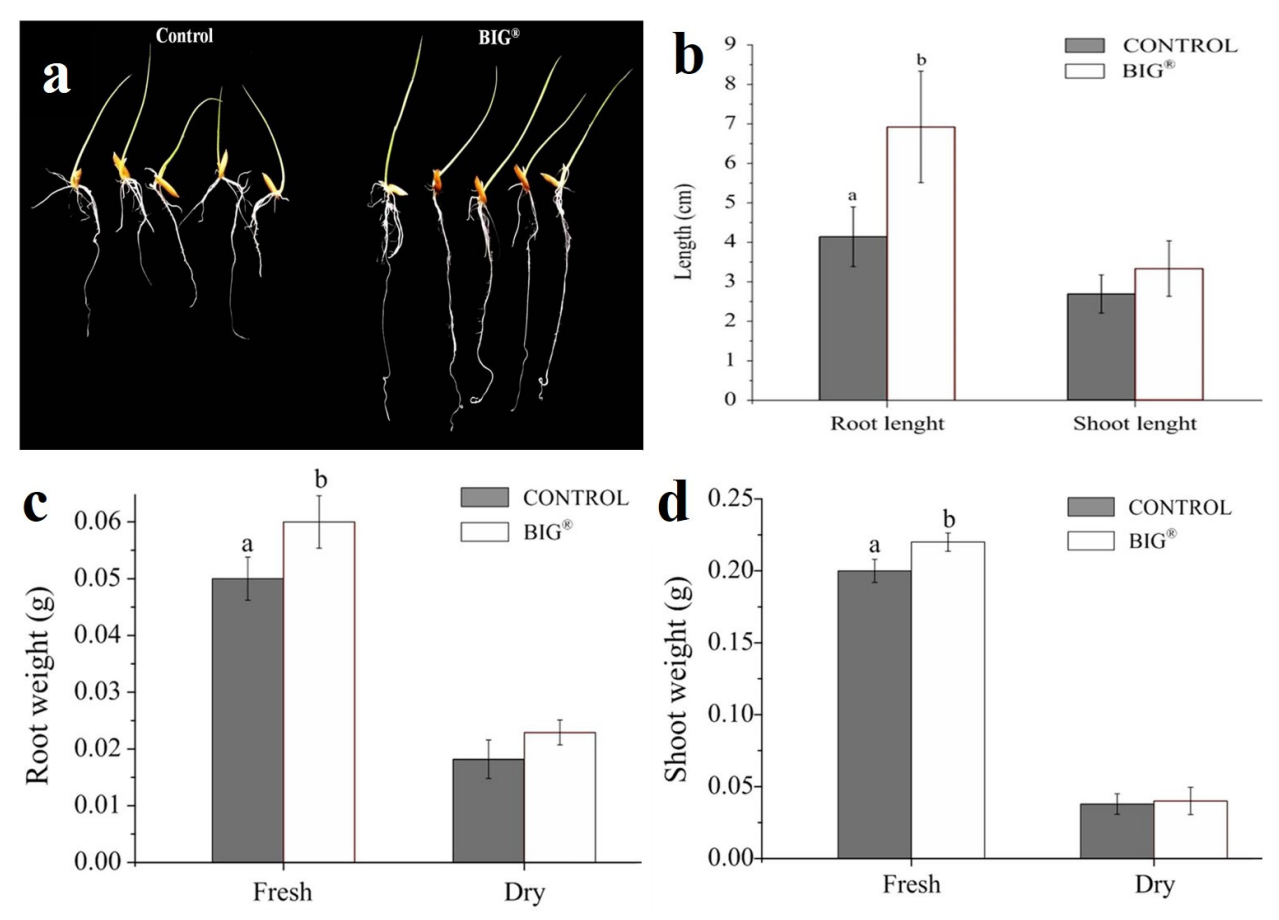
Effect of the natural plant elicitor BIG^®^ on rice growth on a sterile agar medium. (**a**) Quantification of root length and shoot length. (**b**) Root fresh weight and dry weight, (**c**) shoot fresh weight, and (**d**) dry weight after 7 days of cultivation.

In laboratory studies, the BIG^®^ natural plant elicitor treatment had a significant impact on rice seeds of cv. KDML 105. In comparison with the control, seed treatment with BIG^®^ resulted in significant improvements in shoot length (11.5%), root length (67.15%), fresh shoots (10.00%), the dry weight of shoots (5.80%), fresh roots (20%), and the dry weight of roots (25.80%) (Figure 2).

### 2.2. Efficacy of BIG^®^ in Rice Against BLB

**Figure 3:**
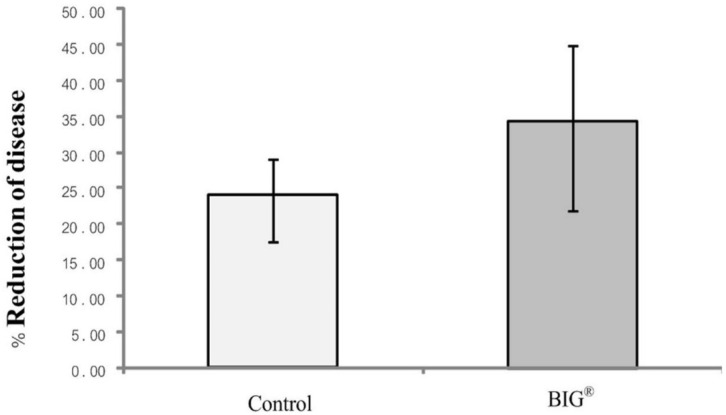
Effect of BIG^®^ on the reduction of rice BLB disease severity under greenhouse conditions.

The BIG^®^ elicitor was evaluated for its ability to induce rice plant resistance to BLB disease when used as a seed treatment in conjunction with foliar spraying every 7 days. The BIG^®^ treatment reduced the severity of BLB by 33% compared with the controls (Figure 3).

### 2.3. BIG^®^’s Biochemical Alterations of Treated Rice Against Xoo

**Figure 4:**
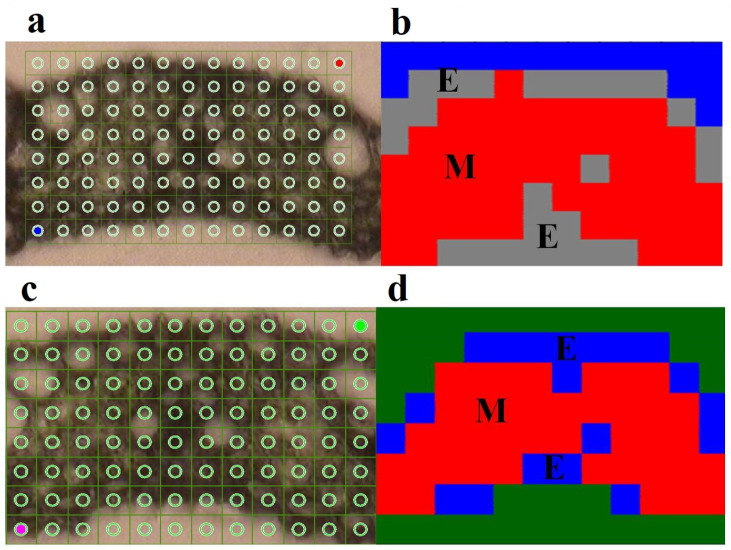
Representative sample of functional group area maps obtained in the spectral region between 1750 and 900 cm^−1^. KDML 105 rice leaves were treated and not treated with the natural plant elicitor BIG^®^ against BLB disease caused by *Xoo.* (**a**) The measurement was performed by means of point-to-point mapping using SR-FTIR microspectroscopy over an area of 130 × 80 μM of rice leaves of cv. KDML 105, were used as a control. (**b**) HCA mapping of rice leaves of cv. KDML 105, used as a control. (**c**) The measurement was performed by means of point-to-point mapping using SR-FTIR microspectroscopy over an area of 120 × 80 μM of rice leaves of cv. KDML 105 treated with the natural plant elicitor BIG^®^. (**d**) HCA mapping of rice leaves of cv. KDML 105 treated with the natural plant elicitor BIG^®^ (E, epidermis; M, mesophyll).

**Figure 5:**
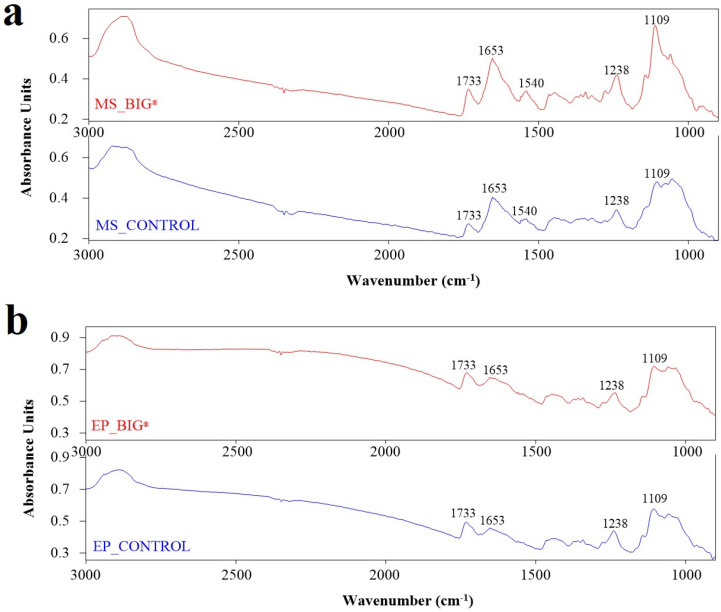
FTIR spectra corresponding to the regions of the mesophyll and epidermis from rice leaves of cv. KDML 105 in response to an inoculation challenge with *Xoo* at 14 DAI under greenhouse conditions. (**a**) Mesophyll of rice plants after inoculation challenge with BIG^®^ compared with the control. (**b**) Epidermis of rice plants after inoculation challenge with BIG^®^ compared with the control.

The biochemical mapping of the rice leaf sections was accomplished by separating specific clusters of the plant’s epidermis (EP) and mesophyll (MS) regions using the SR-FTIR process (Figure 3). The SR-FTIR mapping revealed spectral differences in rice leaves that had not been treated (Figure 4a,b) and those that had been treated with BIG^®^ (Figure 4c,d). Based on the biochemical variability of rice leaves, the HCA model was used to classify the FTIR spectra. Figure 5 depicts the initial mean frequency of the spectral cluster after a challenge with the *Xoo* suspension (Figure 5a,b). Variations in the biochemical alterations of leaf tissues, including cellulose, lignin, pectin, lipids, and mesophyll hemicelluloses, were measured. When compared with non-treated rice leaf epidermis, BIG^®^-treated leaf epidermis showed more variations in biochemical strength.

BIG^®^-treated rice spectra revealed a clear difference between 1513 and 1737 cm^−1^, revealing information about the C=C aromatic ring of lignin and pectin. The conformational changes at 1540 and 1656 cm^−1^ in BIG^®^-treated rice also revealed details on the N-H bending mode of Amide II and the C=O stretching mode of Amide I. Typically, spectral variations were observed in the ranges of 1200–900 and 1240 cm^−1^, which correspond to hemicellulose and C-O-C for cellulose in BIG^®^-treated samples.

**Figure 6:**
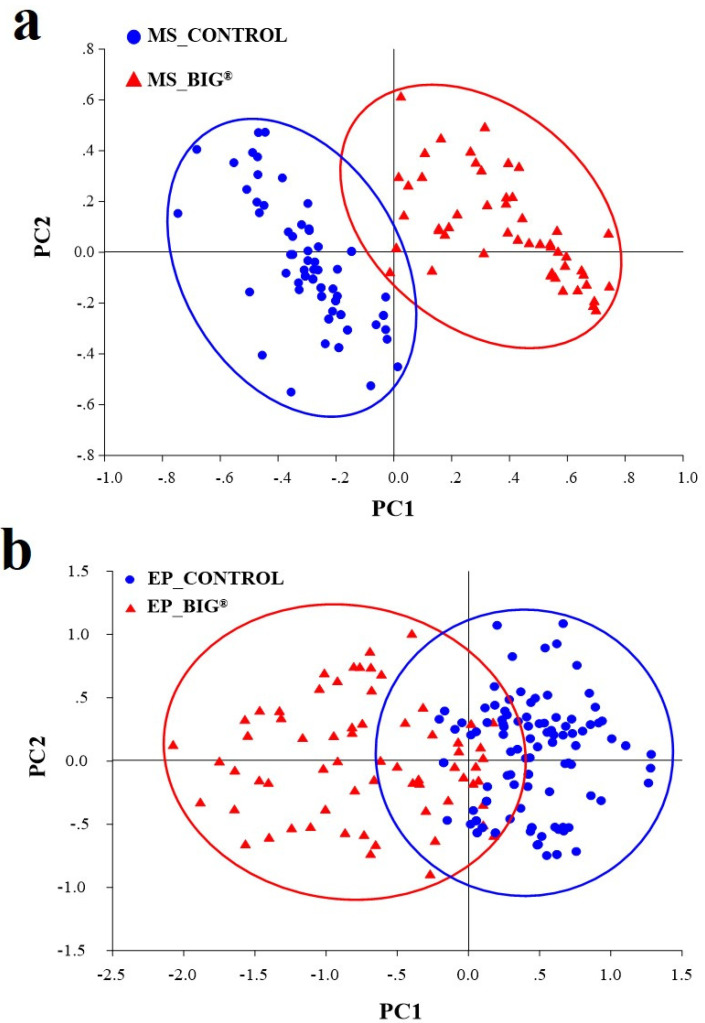
PCA analysis of the mesophyll and epidermis on rice leaves of cv. KDML 105 in response to an inoculation challenge with *Xoo* at 14 DAI under greenhouse conditions. (**a**) Mesophyll of rice plants after inoculation challenge with BIG^®^ compared with the control. (**b**) Epidermis of rice plants after inoculation challenge with BIG^®^ compared with the control.

**Figure 7:**
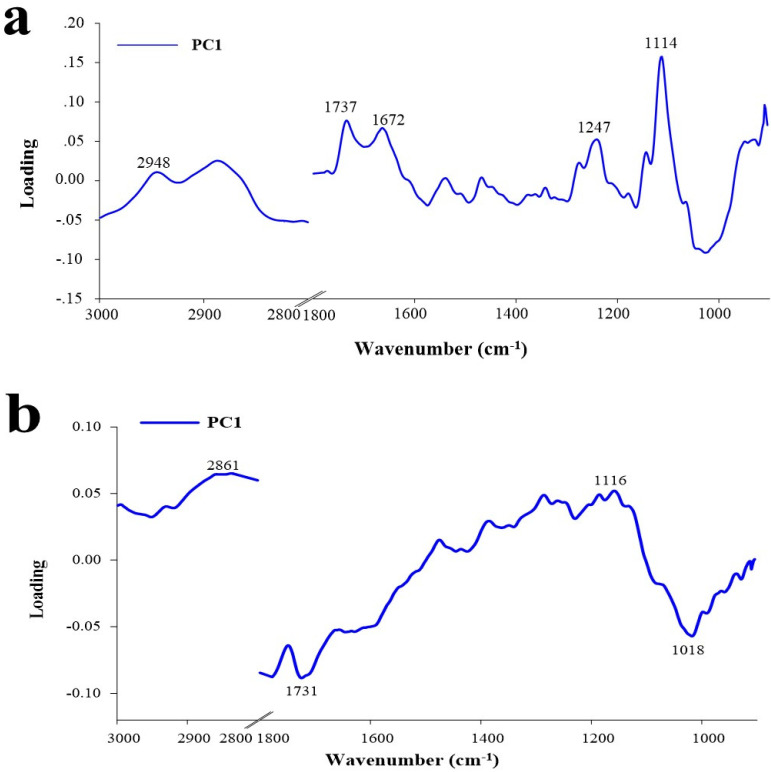
PC1 and PC2 loading plots from PCA analysis on the range of 1800–900 cm^−1^ of the mesophyll and epidermis of rice leaves of cv. KDML 105 in an inoculation challenge with *Xoo* at 14 DAI under greenhouse conditions. (**a**) Mesophyll of rice plant leaves. (**b**) Epidermis of rice plant leaves.

Figure 6 depicts multivariate statistical data analysis strategies using the PCA model (Figure 6a,b). According to these findings, the PCA model was used to statistically evaluate the relevant spectral data for the BIG^®^-induced rice leaves challenged with the BLB pathogen. The first main variable (PC1) showed the highest percentage of spectral variance, followed by PC2. The PC1 and PC2 loads of MS and EP tissues accounted for 69% of the overall variability (PC1 with 50% and PC2 with 19%) and 73% (PC1 with 53% and PC2 with 20%), respectively. Loading plots were used to highlight the biochemical contributions of rice, to define the spectral band variability associated with the second derivative of the average spectra, and to analyze the score plot. The changes in the composition of the BIG^®^-treated biochemical rice compounds revealed that the 1672 and 1737 cm^−1^ parts correspond to the C=O ester of lignin, lipid, and pectin; similarly, the 1114 and 1243 cm^−1^ parts correspond to lignin, cellulose, and hemicellulose (Figure 7a), whereas the influential peak of the PC1 loading from the epidermis corresponded to hemicellulose, cellulose (1018 cm^−1^), and pectin (1731 cm^−1^) (Figure 7b).

**Figure 8:**
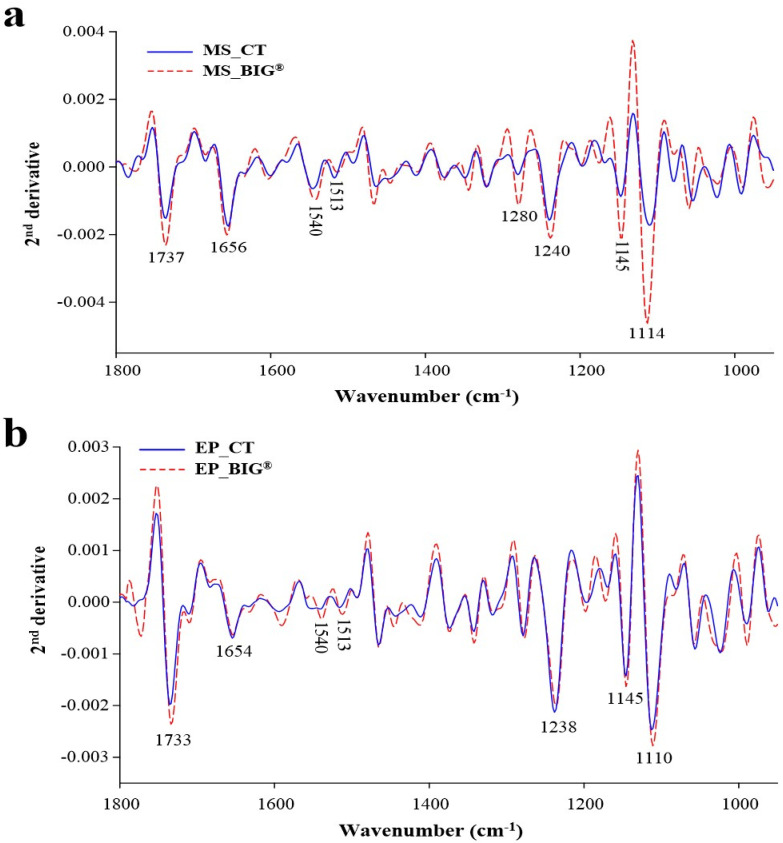
Representative second derivative average spectrum of the mesophyll and epidermis in rice leaves of cv. KDML 105 in an inoculation challenge with *Xoo* at 14 DAI under greenhouse conditions. (**a**) Mesophyll second derivative spectrum of rice after inoculation challenge with BIG^®^ compared with the control. (**b**) Epidermis second derivative spectrum of rice after inoculation challenge with BIG^®^ compared the control.

**Figure 9:**
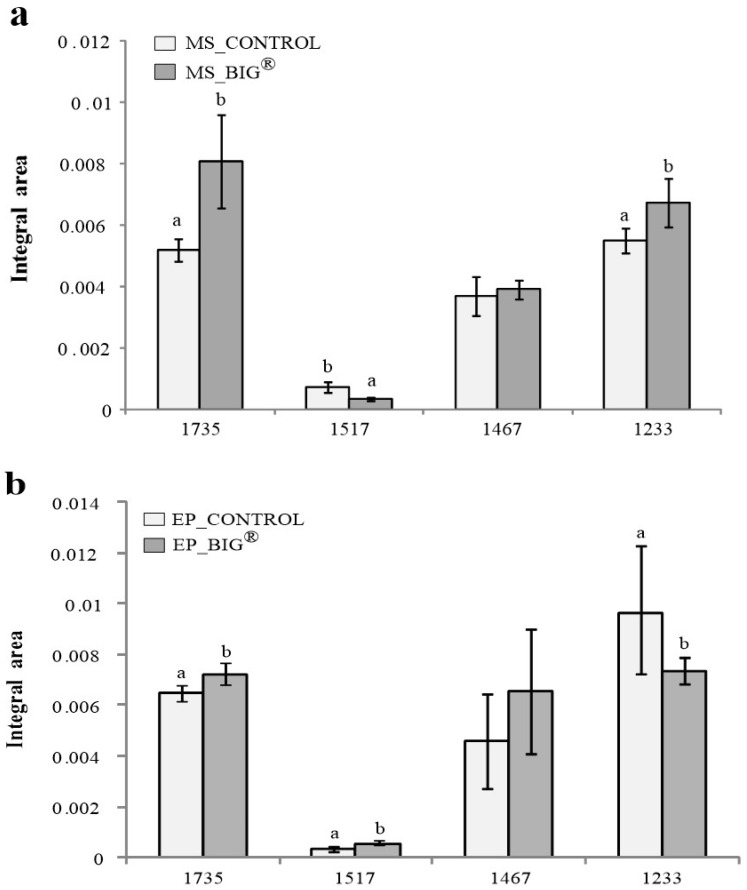
Integral areas of absorbance between 1750 and 900 cm^−1^ of the mesophyll (**a**) and epidermis (**b**) of rice leaves of cv. KDML 105 in an inoculation challenge with *Xoo* at 14 DAI under greenhouse conditions (ρ < 0.05). (**a**) Mesophyll integral areas of rice plants after inoculation challenge with BIG^®^ compared with the control. (**b**) Epidermis integral areas of rice plants after inoculation challenge with BIG^®^ compared with the control.

The average spectral strength allowed for a quantitative analysis of rice leaf tissues, and the different vibrational modes were identified based on the nature of the molecular structures of the biochemical compound (Figure 8a,b). Figure 9a,b shows the integral areas of the biochemical portion of the rice leaf tissues ranging from 1000 to 1800 cm^−1^. The present study revealed that BIG^®^-treated rice showed significantly increased amounts of hemicellulose, cellulose, and pectin in rice mesophyll tissues (*p* < 0.05). The lignin content of the rice leaf epidermis was also increased after BIG^®^ treatment.

### 2.4. Structural Analysis of the Drug Target

**Figure 10:**
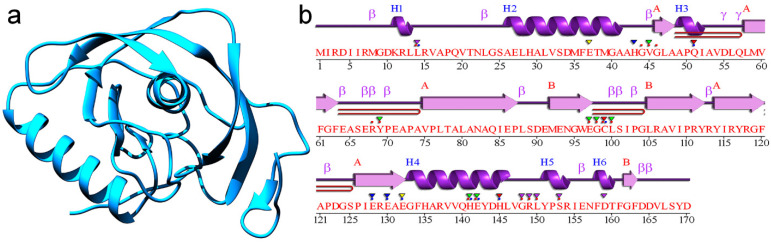
(**a**) 3D molecular structure of peptide deformylase; (**b**) secondary structure of peptide deformylase.

Bacterial leaf blight is one of the most serious bacterial diseases that can devastate the crop when environmental conditions favor the development of diseases. Peptide deformylase (Figure 10a) is a crucial enzyme for bacterial growth and it catalyzes the removal of the N-formyl group from the N-terminal methionine. Several studies have reported on peptide deformylase as a potent target for inhibiting bacterial growth. Hence, to check whether the presence of chitooligosaccharides may be responsible for plant growth and the reduction of BLB severity, chitooligosaccharides were selected as the ligand molecules and peptide deformylase as the target for the molecular docking analysis. Protein structure analysis showed a secondary structure of 2 sheets, 4 beta hairpins, 3 beta bulges, 8 strands, 6 helices, 3 helix–helix interactions, 16 beta turns, and 2 gamma turns (Figure 10b). Additionally, the complex fragment 244, bound in the active site, is shown in Figure 10 and its surface view is presented in Figure 10. The active site is highly focused in the middle of the protein, which shows that the binding of new molecules or drugs can be targeted to this location.

### 2.5. Molecular Interactions—Protein and Polymer Interactions

**Figure 11:**
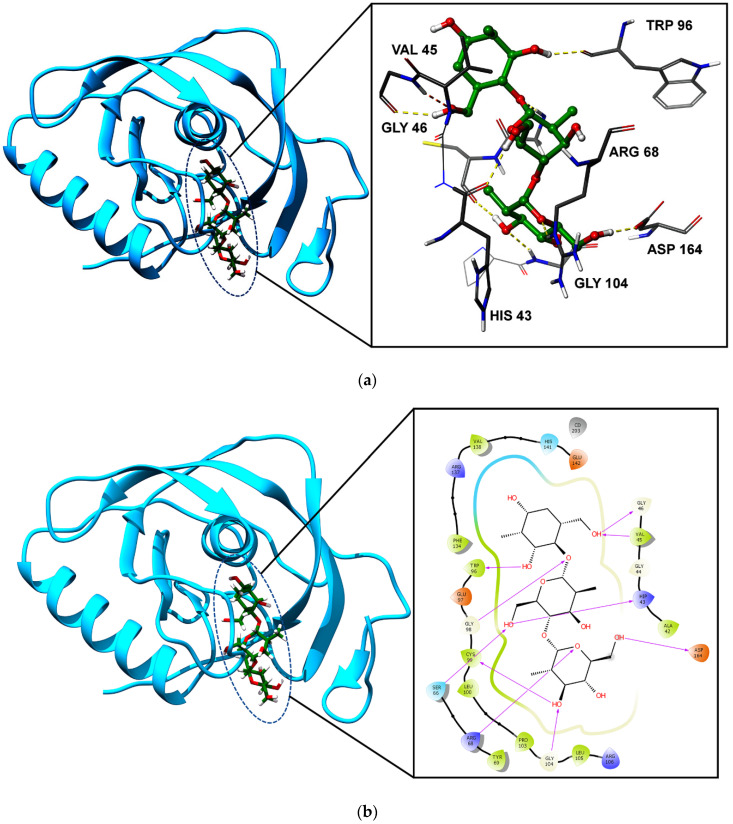
(**a**) 3D representation of protein–polymer interactions, showing ligands in a ball and stick model and core active sites in a stick model, surrounded by a ribbon model. (**b**) Two-dimensional representation of protein–polymer interactions, showing amino acids with a charge-based color model.

Peptide deformylase is a crucial enzyme for bacterial growth and it catalyzes the removal of the N-formyl group from the N-terminal methionine. For this reason, the structural information of PDB was downloaded and prepared for this study. For molecular interactions, the complex bound peptide was investigated for co-crystal complex interactions. Those residues were analyzed for their interactions with the polymer in terms of docking interactions; in association with this, the protein–polymer interactions were analyzed through the IFD docking method. The polymer–protein docking results were interesting, with an IFD score of −327.62 kcal/mol, while the re-docked pose of the native ligand (56V), shows an IFD score of −298.38 kcal/mol. The native bound compound and the polymer-bound compound in the protein’s active site in similar binding modes showed the RMSD value of 0.218Å. For the native ligand molecules, only Tyr69 was able to form the hydrogen bond interaction (Figure S1). However, the interactions between the protein and the polymer showed strong binding with 10 hydrogen bonds, especially with the active site residues His43, Val45, Gly46, Ser66, Arg68, Trp96, Gly98, Cys99, Gly104, and Asp164. Representations of the protein–polymer docking interactions in two and three dimensions are presented in Figure 11a,b. The interaction profiles for the native ligand and the polymer with the protein showed that strong interactions are seen with the polymer than with the native ligand molecule. This may be due to the smaller number of atoms available in the native ligand with an aromatic ring feature, while the polymer molecule has more atoms; in addition, the polymer molecule has more hydrogen bond acceptors and donor features, along with the aromatic ring feature. This causes the polymer to form strong hydrogen bond interactions with the protein, in comparison with the known native ligand molecule. The interaction profiles were also matched with the predicted binding energy (MM/GBSA calculation), showing −39.09 kcal/mol for the native molecule, while the polymer showed a binding energy of −66.82 kcal/mol. MM-GBSA binding energies are approximate free energies, and a molecule which emits lower binding energy, i.e., a more negative value, indicates stronger binding. Through this, the polymer molecule shows strong binding than the native compound in terms of energy, and the interactions were evaluated.

### 2.6. Quantum Mechanical Calculations

**Figure 12:**
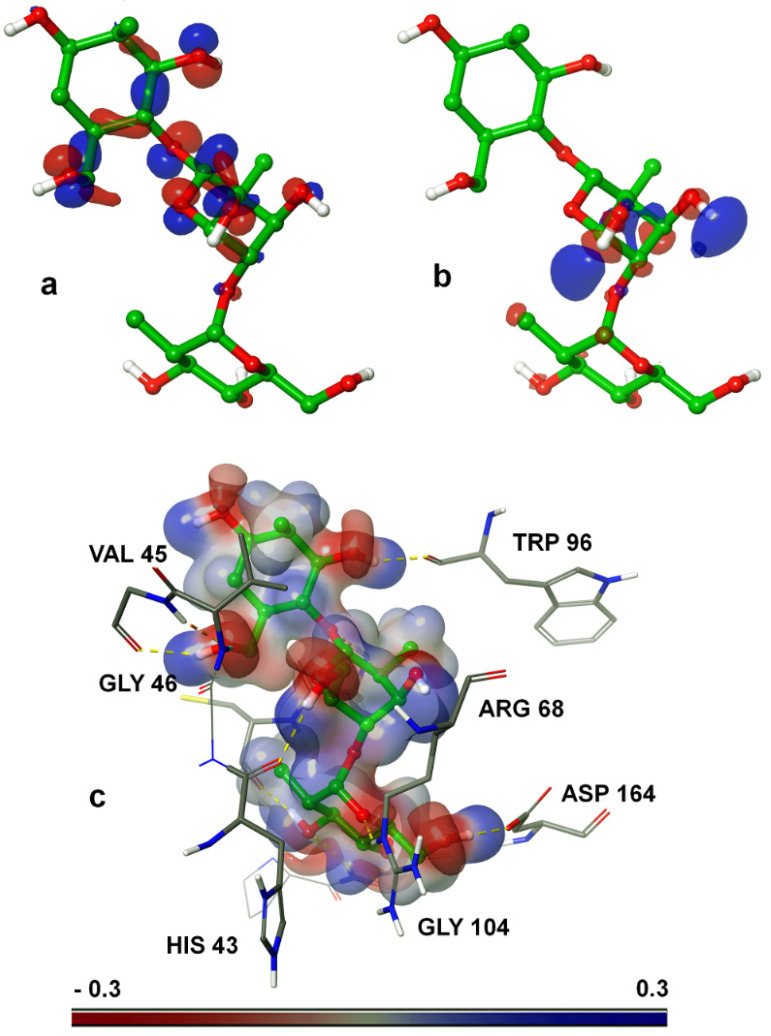
QM-based HOMO/LUMO and MESP charge calculations for the protein and polymer interaction complex. (**a**) Representation of the HOMO, (**b**) representation of the LUMO, and (**c**) representation of the MESP charge in the interval of −0.3 to 0.3 level charges.

The electron transfer capacity between the protein’s active site and the polymer was calculated using quantum mechanical calculations. This was performed to understand the electronic properties of the polymer’s structure in the bound state. For this, the highest occupied molecular orbital (HOMO), lowest unoccupied molecular orbital (LUMO), and molecular electrostatic potential (MESP) were calculated for the bound conformation. For the polymer, the MESP, HOMO/LUMO parameters, free energy, and solvation energy were analyzed. The bound conformation of the polymer showed HOMO and LUMO energies with minimal values of 0.243 and 0.182 eV, respectively. Representations of the 3D surface based on the HOMO/LUMO and MESP calculations were plotted onto the polymer, as shown in Figure 12. Figure 12a shows the HOMO surface, Figure 12b shows the LUMO surface, and Figure 12c shows the MESP surface. The chitooligosaccharides have a cationic charge, with Figure 12b showing that the lowest unoccupied molecular orbital is located in the middle region, and with Figure 12a showing that the highest occupied molecular orbital was located just above the LUMO surface. For the MESP, we have depicted the positive surface in blue, the negative surface in red, and the neutral surface in white in Figure 12c, showing that the polymer holds a high positive charge on the surface and limited negative charges, with a much lower number of neutral charges. These positive and negative charges show the interactions with negatively and positively charged atoms, respectively. The results thus show that physical interactions occur between the protein and polymer, forming a complex, showing the positively charged atoms’ interactions with negatively charged atoms and vice versa. The charge density of the MESP was calculated in the range between −0.3 and 0.3 for the negative and positive charges, and their surface alignments with the charges are shown in Figure 12c. Due to this charge allocation function between the protein and the polymer, the polymer molecule tended to share the donor and accept the acceptors in the His43, Val45, Gly46, Ser66, Arg68, Trp96, Gly98, Cys99, Gly104, and Asp164 amino acids, resulting in the formation of 10 hydrogen bonds, which, in turn, may have inhibited the binding potential.

### 2.7. Molecular Dynamics Simulation

**Figure 13:**
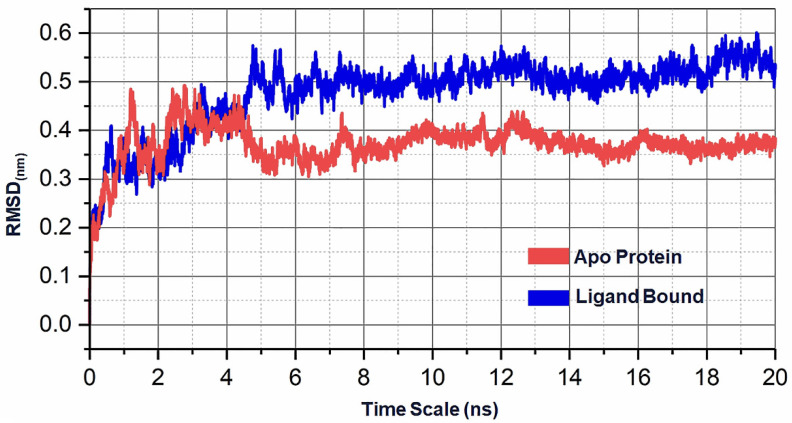
RMSD chart showing a representation of the deviation that occurred within 20 ns in reference to the initial structure for both apo (red) and holo (blue) forms.

An MD simulation was conducted for the apo (*Xoo*1075) and holo forms (*Xoo*1075-bound chitooligosaccharides) at the timescale of 20 ns to understand the stability of the protein in its apo and holo forms and also to understand its dynamic behavior. For both the apo and holo forms, the MD simulations were performed in a similar environment in order to understand the differences between the forms. The illustration presented in Figure 13 shows the dynamic values plotted in reference to the initial structure. The apo form showed initial deviations of up to 0.48 nm up to the fourth nanosecond and reached a stable level around 0.38–0.42 nm. In terms of its average deviation across the entire 20 ns timescale, the apo form showed ~0.37 nm. Similarly, the ligand-bound holo form showed similar deviations up to the fourth nanosecond and a shift occurred after the fourth nanosecond that led it to be positioned above ~0.5–0.6 nm throughout the MD simulations; on average, the holo forms showed deviations of ~0.47 nm. The difference between the apo and holo forms is clearly shown in the RMSD graph, showing that higher deviations occurred in the holo forms, due to the atomic interactions between the protein and polymers, or conformational changes of the polymer that occurred up to the fourth nanosecond and stabilized during the 20 ns MD simulation.

## 3. Discussion

In agricultural production, chitin, chitosan, and COS are potential biocontrol elicitors [12]. In the current study, we found that a COS elicitor, BIG^®^, was able to boost growth and control disease in rice of cv. KDML 105. Tanaka et al. reported a similar result in their previous analysis [13]. In addition, chitin and its oligomers have been identified as effective elicitors in a variety of plants. As phytopathogens invade the hosts, elicitors for plant defense reactions are activated, including the processes of inhibitors, hydroxyproline-rich glycoproteins, active oxygen species, phytoalexins, enzymes, lignification, and proteinase. In this study, the role of the plant elicitor BIG^®^ in regulating BLB and its mode of action was tracked by observing changes in the biochemical components. Based on the SR-FTIR results, BIG^®^ was found to potentially mediate the ability of rice to resist BLB, with a damage reduction of approximately 33%.

BIG^®^ may elicit defense responses involving changes in several defense-related biochemical compositions, thereby increasing resistance to BLB disease in rice plants. In a previous study, COS molecules were used to induce defense responses in rice plants against blast disease. Mechanisms such as the increased activity of the phenylalanine ammonia lyase enzyme, programmed cell death, and H_2_O_2_ were observed under COS treatment compared with the control treatment, which resulted in a reduction in visible symptoms [14]. The activation of systemic resistance within host plants may be responsible for the reduction in disease incidence in COS-produced rice. According to Reglinskiet et al., COS significantly reduced the incidence and severity of rice leaf streaking in greenhouse conditions. COS’s main antibacterial activity may be to increase the activity of resistance enzymes such as peroxidase, PAL, and polyphenol oxidase [15]. The resistance activity of these enzymes causes changes in biochemical components that can be detected using the FTIR technique.

The vibrations of the C–O bonds of cellulose, hemicellulose, polysaccharides, and protein groups were shown to form FTIR spectra in the typical carbohydrate area (from 800 to 1200 cm^−1^) from tissues and plant cells. The stretching of C=O, C-C, and N-H could be assigned to mesophyll and vascular bundles that had a protein amount of 1500–1700 cm^−1^. The C=C stretching corresponded to 1513 cm^−1^, which is assigned to lignin. The band attributed to pectin, primarily from C=O stretching, was discovered at 1737 cm^−1^ [16]. The biochemical components in the rice leaf tissues were detected using SR-FTIR microspectroscopy to describe the defense mechanism within the BIG^®^-treated rice plant. The current study found that SR-FTIR spectral changes in the mesophyll and epidermis indicative of stretch changes were observed in BIG^®^-induced rice leaf tissues, followed by the *Xoo* inoculation challenge. Peaks 1656, 1540, 1513, and both 1513 and 1737 cm^−1^ indicated C=O stretching of the helix protein, N–H bending and C–N stretching indicated of the protein, the C=C indicated aromatic ring of lignin, and the C=O ester of the lipid bond, pectin, and lignin, respectively. The spectral range of 1200–1000 cm^−1^, which corresponded to the C–C cellulose ring and C–O stretching of hemicellulose, was significantly expanded (Figure 8a,b).

The resistance mechanism indicated that the defensive mechanisms involved in protecting plant cell walls had contributed to the accumulation of pectin and lignin [10,17]. To prevent pathogen colonization in the cell, phenolic polymers (lignin or lignin-like) are synthesized and rapidly produced in cell walls. In addition, lignin accumulated in infected plant cells may reduce the spread of phytopathogen toxins and enzymes. After *Xoo* inoculation, the intensity of lignin (1513 cm^−1^) in BIG^®^-treated leaves was higher. A high level of lignin (1513 cm^−1^) may improve the mechanical strength of the cell wall, such as its thickness or toughness, and make it more resistant to pathogens [18]. The plant’s susceptibility to disease and the pathogen’s mechanism has previously been described. Spectrum variations in epidermis leaf tissue suggested higher integral areas for the C=O ester from polysaccharides (900 to 1200 cm^−1^), as well as pectin, lignin, or lipids (1700 to 1770 cm^−1^) in chili plants pre-treated with *B. subtilis* strain D604, then inoculated with *C. acutatum* [19].

BIG^®^-induced protein accumulation in inducible plants is also intriguing. Previous studies have explained that after pathogen stimulation, the activation of enzymes that can change the plant’s morphology, such as enzymes that work to reshape the cell wall or catalyze enzymes that damage the pathogen, was induced [20]. The use of SR-FTIR microspectroscopy to characterize the structures of secondary metabolites inside plants has become increasingly important in the host–elicitor interaction. Thumanu et al. discovered that alpha helices (Amide I protein with a peak of 1650 cm^−1^) could not be transformed into beta sheets (1600 cm^−1^) in the mesophyll tissues of chili plants that were not caused by D604 [19]. According to the findings of this study, BIG^®^ (at 2 mL/L) was shown to induce changes in plant cells related to activating enzymatic activity and cell wall lignification. Furthermore, lignin is primarily composed of sinapyl alcohol monolignols and coniferyl, which contribute to the formation of syringyl polymer and guaiacyl units. BIG^®^ induced a band amplitude at 1470 cm^−1^ that was correlated with C–H bending in the alkyl groups of lignin syringyl. Higher levels of syringyl units were correlated with phenylalanine ammonia lyase activity, according to Cass et al. (2015) [21]. COS has been shown to modulate plant reactions in a variety of plant systems, including the induction of defense-related phytoalexin, lignification, and PR-proteins; the activation of callose and cell wall formation; and the development of proteinase [22]. The epidermis and mesophyll tissues of BIG^®^-treated rice plants exhibited increasing SR-FTIR spectra, which corresponded to changes in the composition of polysaccharides, proteins, lipids, lignins, and rice leaf pectins.

The target *Xanthomonas oryzae* pv. *oryzae* enzyme, *Xoo* 1075 (PDB ID: 5CY8), has been studied for its interactions with polymer ingredients, specifically chitooligosaccharides, to gain an atomic-level understanding of the molecular processes involved. The findings are intriguing, with the chitooligosaccharide polymer ingredients binding strongly to the drug target and revealing the presence of 10 hydrogen bonds between the protein and the polymer, among other observations. High-end QM methods revealed that chitooligosaccharides have fewer LUMO regions than other ingredients, whereas the HOMO regions are highly occupied, resulting in strong electron transfer between the protein and the polymers. Furthermore, MESP shows evidence of strong interactions between the protein and polymer, as opposed to the opposite-charge-based interactions seen in other studies. Overall, the computational methods supported the experimentally demonstrated strong binding of chitooligosaccharides to the drug target. The polymer’s molecular structure contains an aromatic ring feature, which shows tight interactions within the active site and shows lower binding affinity. This polymer, in dynamic equilibrium, showed moderate fluctuations up to the fourth nanosecond and, when the interactions became stable between the protein and polymer, the holo forms became stable throughout the 20 ns of the MD simulation process. Overall, this study confirmed the strong binding of the polymer and also demonstrated that the polymer was stable throughout the MD simulation process.

## 4. Materials and Methods

### 4.1. Preparation and Cultivation of Elicitor and Bacterial Pathogen

Rice (*Oryza sativa L.)* seeds that were susceptible to BLB disease (cv. KDML 105), a natural plant elicitor with active ingredient-based chitooligosaccharides (BIG^®^), and a bacterial pathogen with high virulence (*Xoo* strain SUT1-121) were used in this study, which was undertaken by the Plant Pathology and Biopesticide Laboratory of Suranaree University of Technology. The culture was activated on a nutrient broth and kept at 28 °C until Day 2. The bacterial colony was mixed with sterile distilled water before being adjusted to 1 × 10^8^ CFU/mL with an absorbance of 0.2 at 600 nm as measured using a NanoDrop Thermo 2000/2000 c spectrophotometer.

### 4.2. Assessing the Efficacy of BIG^®^ on Rice Growth

The rice seeds were surface-treated with ethyl alcohol (EtOH) for approximately 30 s before being immersed 3 times in sterile water. The rice seeds were then soaked in a BIG^®^ solution at a concentration of 2 mL/L for 1 h. Instead of the BIG^®^ solution, the control rice seeds were soaked in sterile distilled water. Four replicates were performed on 4 plates for each treatment, with 10 seeds per plate (100 × 15 mm in size). The rice seeds were incubated in Petri plates containing 1% sterile agar under laboratory conditions, with a 16/8 h light/dark cycle and day and night temperatures of 27 °C and 24 °C, respectively. A sample of enhanced rice growth was taken 7 days after seed germination. On Day 7, the length of the root and shoot, as well as the weight of the shoot and root (dry and wet), were measured and recorded [23]. The experiment was carried out in a completely randomized design (CRD) with 2 treatments, 4 replicates and repeated 3 times.

### 4.3. Greenhouse Studies

The experiment was conducted in CRD with two treatments, four replicates and repeated 3 times. Sterile seed surfaces were obtained in a similar way to the abovementioned methodology. After soaking in the BIG^®^ solution at 2 mL/L for approximately 1 h, the rice seeds were incubated in Whatman moisture testing paper in dark conditions [24]. Next, the rice seedlings were transferred to pots for greenhouse studies. Every 7 days after sowing (DAS), the rice plants were sprayed with the BIG^®^ solution (2 mL/L) until surface leaf runoff was observed. The procedure was carried out in the same manner with sterile distilled water in control or untreated plants [4].

### 4.4. Xoo Pathogen Inoculation

Before bacterial pathogen inoculation, rice plants were treated with BIG^®^ or sterile distilled water in the controls. As previously described, a suspension of *Xoo* strain SUT1-121 at 1 × 10^8^ CFU/mL was prepared. The rice plants were inoculated with the *Xoo* suspension at 45 DAS via foliar spraying and cutting/dipping methods. The rice plants were covered with plastic bags for 1 day after *Xoo* inoculation to maintain moisture. Disease reduction was measured by calculating the infection of the rice leaf. Four replicates and three repeats were used in the experiment [25].

### 4.5. Detection of Biochemical Changes in Rice Leaves Using SR-FTIR Microspectroscopy

#### 4.5.1. Preparation of Rice Leaves for Analysis

The rice leaf samples were thinned to 7 μM before being scanned for SR-FTIR microspectroscopy. In all treatments, rice leaf samples were collected 7 days after the *Xoo* challenge. They were cut into 4 × 8 mM pieces and immediately coated with the Tissue-Trek optimum cutting temperature (OCT) compound (Electron Microscopy Sciences Inc., Hatfield, USA) and frozen. Until cryosectioning, each sample was kept at −80 °C. Through use of a Microm HM 525 Cryostat (Thermo Fisher Scientific), the sample was cut into small pieces with a thickness of 7 μM and the full leaf structure (epidermis and mesophyll) was preserved. The frozen rice leaves were placed on a BaF2 window, which is an infrared transparent material. To avoid the influence of water peaks in the examination, the water moisture in the sample was evaporated using a desiccator. The SR-FTIR spectra were chosen to be in the mid-infrared range [26].

#### 4.5.2. SR-FTIR Microspectroscopy Analysis

The IR spectra (4000 to 800 cm^−1^) of the sample were obtained and collected using a system that included the 36× objective lens of an infrared microscope (Hyperion 2000, Bruker), an SR-FTIR (Hyperion 2000, Bruker Optics, Ettlingen, Germany) system, and a liquid nitrogen-cooled MCT D315 detector. To record 64 scans, a spectral resolution aperture (1010 M) of 4 cm^−1^ was used in conjunction with the mapping mode. To monitor and analyze the data, CytoSpec version 1.3 (Cytospec Inc., NY, USA) and OPUS version 7.2 (Bruker Optics Ltd., Ettlingen, Germany) were used. Analytical models based on IR spectra were used to clarify the biochemical differences in rice plant tissues between BIG^®^-treated and non-treated samples, particularly in the epidermis and mesophyll tissues. The second derivative was generated using 13 smoothing points, and the effects of different sample thicknesses were normalized vectors. For a univariate model, the peak strength, peak area, and peak yield ratio were used for chemical mapping or feature group mapping. An HCA model based on SR-FTIR data with spectral ranges between 3000 and 2800 cm^−1^ and 1800 and 900 cm^−1^ was used to characterize the biochemical components. The HCA graph was generated using SR-FTIR mapping over a region of 130 × 80 μM for the untreated rice and 120 × 80 μM for the BIG^®^-treated rice. Clustering algorithms were used to create a 2D image file with a unique color for each cluster [27]. The biochemical components of mesophyll leaf tissues were differentiated using PCA in multivariate data analysis models based on the spectra of each cluster. Unscrambler X version 10.1 (CAMO, Norway) software, with the function “Savitzky–Golay method” with the third polynomial and 13 smoothing points, was used to calculate the second-derivative spectra. Data analysis was carried out at BL4.1 Infrared Spectroscopy and imaging at the Synchrotron Light Research Institute (SLRI), Nakhon Ratchasima, Thailand, using licensed software tools.

### 4.6. Protein and Polymer Preparation

The protein enzyme *Xoo*1075 from *Xanthomonas oryzae* pv. *oryzae* (PDB ID: 5CY8) was used to understand the molecular processes underlying chitooligosaccharide (polymer) binding, which directly helps in promoting growth and resistance to BLB. For this purpose, the protein and polymer substances were placed into 3D coordinates to prepare for their adoption in the molecular modeling environment [28]. Initially, the protein structure was downloaded from a protein data bank and imported in Schrodinger Maestro, and the missing residues and side chains were refined, along with hydrogen bond optimization. The optimized protein was subjected to minimization until the protein reached an RMSD value of 0.30 Å. Similarly, the polymer chitooligosaccharides were drawn using Chemdraw, and the 2D structure was converted into a 3D structure using LiGprep [29]. The OPLS-AA forcefield was applied for the conversion and preparation processes, with an output of 32 conformations per ligand.

### 4.7. Induced Fit Docking

The prepared protein and polymer were subject to molecular docking using the induced fit docking (IFD) method [30]. The IFD method uses flexible ligands and flexible protein conformations for docking. Through this process, highly accurate conformations can be obtained. The ligand-binding position is marked with the available co-crystal ligand bound with the protein, which is replaced in the polymer through the molecular docking method. Active sites are marked for docking interactions, and side chains are remodeled through priming [31]. Through this process, the bound interactions of the polymer can be obtained with high accuracy and realistic interactions. The energy between the complexes was calculated using the prime MM/GBSA method for calculation of the binding energy, based on the final protein–polymer complex [32].

### 4.8. Quantum Mechanical Calculations

The electronic charges observed in the protein–polymer electron transfer process can be used to elucidate the pharmacological aspects that will be found through DFT analysis [33]. In this process, the ligand from the IFD process was separated from the complex and imported using the Jaguar workflow. To assign the functional set, B3LYP with 6-31G**++ was used, along with the basic set function [34]. Multiple moments were assessed with the coupled perturbed Kohn–Sham (CPKS) equations, and for each element, the Poisson–Boltzmann finite (PBF) equation was used. For the results, the QM properties of the polymers were calculated to understand the molecular orbitals, using HOMO, LUMO, and MESP calculations [35].

### 4.9. Molecular Dynamics Simulations

A molecular dynamics (MD) simulation was performed for the protein structure of *Xoo*1075 from *Xanthomonas oryzae* pv. *oryzae* (PDB ID: 5CY8) in 2 forms: the apo form and the holo form (the chitooligosaccharide bound form), using GROMACS 2018.1 [36]. Both the apo and the holo form were subjected to system preparation with an OPLS-AA force field and a TIP3P water model, packed inside the periodic boundary box, with a distance of 1 nm between the structure and the cubic box [37,38]. For the holo form, the ligand topology was obtained externally from the PRODRG server (http://davapc1.bioch.dundee.ac.uk/cgi-bin/prodrg (accessed on 20 June 2021)) and integrated with the protein topology in the MD system box [39]. For minimization of the system, the appropriate number of ions was added, based on balancing the charge of the system box, and the initial minimization was performed for both the apo and holo forms, with up to 1000 steps of minimization, using the steepest descent algorithm with a tolerance of 10 kJ/mol/nm to clear the steric clashes [40,41]. The temperature was maintained, with 300 K as the reference room temperature, using thermostat coupling with a Berendsen thermostat and pressure coupling with a pressure of 1.0 using a Parrinello–Rahman barostat, along with periodic boundary conditions with a cut-off for Coulomb interactions and the Lennard–Jones potential [42]. The long-range interactions of biomolecular systems were calculated using the particle mesh Ewald method, and minimization was performed for the whole system for 1000 ps, with 300 K and 1 bar of pressure with both the NVT and NPT ensembles. The final well-equilibrated minimized system was subjected to an MD run for the timescale of 20 ns and RMSD analysis for both the apo and holo forms [43].

### 4.10. Statistical Analysis

SPSS software version 18 was used to analyze the data for each experiment and the analysis of variance (ANOVA). In addition, the significant difference between treatments was determined using Duncan’s multiple range test (DMRT) at *p* < 0.05.

## 5. Conclusions

The findings of this study showed that BIG^®^ was able to promote rice plant growth, while also activating BLB resistance. The use of microscopy and spectroscopy, which measured changes in the biochemical components within plant tissues, provides a novel approach to understanding plant responses to environmental stimuli. Furthermore, when combined with multivariate statistical techniques, SR-FTIR microspectroscopy may be able to provide information on the function of biochemical components in host plant defense mechanisms. In addition, high-throughput approaches to BLB monitoring, such as transcriptomics and proteomics analyses, can provide additional guidance for the study of these mechanisms. High-end QM methods revealed that the chitooligosaccharide ingredients of polymers had fewer LUMO regions than other ingredients, whereas the HOMO regions were highly occupied, resulting in strong electron transfer between the protein and the polymers. Furthermore, MESP analysis showed evidence of strong interactions between the proteins and polymers that occurred through opposite-charge-based interactions between the proteins and polymers. These findings shed new light on the relationship among BIG^®^, rice, and the plant disease-resistance mechanisms.

## Data Availability

The data used to support the findings of this study are available from the corresponding author upon request.

## Supplementary Materials

The following are available online at https://www.mdpi.com/article/10.3390/ijms22157990/s1.

## References

[1] Xu J., Audenaert K., Hofte M., De Vleesschauwer D. (2013). Abscisic acid promotes susceptibility to the rice leaf blight pathogen *Xanthomonas oryzae* pv. *oryzae* by suppressing salicylic acid-mediated Defenses. PLoS ONE.

[2] Le Thanh T., Thumanu K., Wongkaew S., Boonkerd N., Teaumroong N., Phansak P., Buensanteai N. (2017). Salicylic acid-induced accumulation of biochemical components associated with resistance against *Xanthomonas oryzae* pv. *oryzae* in rice. J. Plant. Interact..

[3] Mandal S., Mallick N., Mitra A. (2009). Salicylic acid-induced resistance to *Fusarium oxysporum* f. sp. *lycopersici* in tomato. Plant. Physiol Biochem..

[4] Buensanteai N., Yuen G.Y., Prathuangwong S. (2009). Priming, signaling, and protein production associated with induced resistance by *Bacillus amyloliquefaciens* KPS46. World J. Microbiol. Biotechnol..

[5] Thepbandit W., Papathoti N.K., Daddam J.R., Thumanu K., Siriwong S., Thanh T.L., Buensanteai N. (2021). Identification of salicylic acid mechanism against leaf blight disease in *Oryza sativa* by SR-FTIR microspectroscopic and docking studies. Pathogens.

[6] El Hadrami A., Islam M.R., Adam L.R., Daayf F. (2015). A cupin domain-containing protein with a quercetinase activity (VdQase) regulates *Verticillium dahliae’s* pathogenicity and contributes to counteracting host defenses. Front. Plant. Sci..

[7] Bastas K.K. (2014). Importance of reactive oxygen species in plant-pathogen interactions. Selcuk J. Agri. Food Sci..

[8] Bouranis D., Chorianopoulou S., Vassilis B., Protonotarios Ε., Siyiannis F., Malcolm B., Hawkesford J. (2007). Localization of reactive oxygen species and lignification in leaves of young sulphate-deprived maize plants. Funct. Plant Sci. Biotechnol..

[9] Garcion C., Lamotte O., Cacas J., Métraux J. (2014). Mechanisms of defence to pathogens: Biochemistry and physiology. Induced Resistance for Plant Defense.

[10] Lahlali R., Song T., Chu M., Yu F., Kumar S., Karunakaran C., Peng G. (2017). Evaluating changes in cell-wall components associated with clubroot resistance using fourier transform infrared spectroscopy and RT-PCR. Int. J. Mol. Sci..

[11] Papathoti N.K., Saengchan C., Daddam J.R., Thongprom N., Tonpho K., Thanh T.L., Buensanteai N. (2020). Plant systemic acquired resistance compound salicylic acid as a potent inhibitor against SCF (SKP1-CUL1-F-box protein) mediated complex in *Fusarium oxysporum* by homology modeling and molecular dynamics simulations. J. Biomol. Struct. Dyn..

[12] Jia X., Meng Q., Zeng H., Wang W., Yin H. (2016). Chitosan oligosaccharide induces resistance to Tobacco Mosaic Virus in *Arabidopsis* via the salicylic acid-mediated signalling pathway. Sci. Rep..

[13] Tanaka K., Cho S.-H., Lee H., Pham A.Q., Batek J.M., Cui S., Qiu J., Khan S.M., Joshi T., Zhang Z.J. (2015). Effect of lipo-chitooligosaccharide on early growth of C4 grass seedlings. J. Exp. Bot..

[14] Pongprayoon W., Roytrakul S., Pichyangkura R., Chadchawan S. (2013). The role of hydrogen peroxide in chitosan-induced resistance to osmotic stress in rice (*Oryza sativa* L.). Plant Growth Regul..

[15] Reglinski T., Stavely F.J.L., Taylor J.T. (1998). Induction of phenylalanine ammonia lyase activity and control of *Sphaeropsis sapinea* infection in *Pinus radiata* by 5-chlorosalicylic acid. Eur. J. Plant Pathol..

[16] Heraud P., Caine S., Sanson G., Gleadow R., Wood B.R., McNaughton D. (2007). Focal plane array infrared imaging: A new way to analyse leaf tissue. New Phytol..

[17] Pogorelko G.V., Kambakam S., Nolan T., Foudree A., Zabotina O.A., Rodermel S.R. (2016). Impaired chloroplast biogenesis in immutans, an Arabidopsis variegation mutant, modifies developmental programming, cell wall composition and resistance to *Pseudomonas syringae*. PLoS ONE.

[18] Yang C., Liang Y., Qiu D., Zeng H., Yuan J., Yang X. (2018). Lignin metabolism involves *Botrytis cinerea* BcGs1- induced defense response in tomato. BMC Plant Biol..

[19] Thumanu K., Wongchalee D., Sompong M., Phansak P., Le Thanh T., Namanusart W., Vechklang K., Kaewnum S., Buensanteai N. (2017). Synchrotron-based FTIR microspectroscopy of chili resistance induced by *Bacillus subtilis* strain D604 against anthracnose disease. J. Plant Interact..

[20] Freeman (2008). An overview of plant defenses against pathogens and herbivores. Plant Health Instr..

[21] Cass C.L., Peraldi A., Dowd P.F., Mottiar Y., Santoro N., Karlen S.D., Bukhman Y.V., Foster C.E., Thrower N., Bruno L.C. (2015). Effects of phenylalanine ammonia lyase (PAL) knockdown on cell wall composition, biomass digestibility, and biotic and abiotic stress responses in *Brachypodium*. J. Exp. Bot..

[22] Benhamou N., Nicole M. (1999). Cell biology of plant immunization against microbial infection: The potential of induced resistance in controlling plant diseases. Plant Physiol. Biochem..

[23] Kalaivani K., Kalaiselvi M.M., Senthil-Nathan S. (2016). Effect of methyl salicylate (MeSA), an elicitor on growth, physiology and pathology of resistant and susceptible rice varieties. Sci. Rep..

[24] Shivalingaiah U.S., Umesha S. (2013). *Pseudomonas fluorescens* inhibits the *Xanthomonas oryzae* pv. *oryzae*, the bacterial leaf blight pathogen in rice. Can. J. Plant Protect..

[25] Melotto M., Panchal S., Roy D. (2014). Plant innate immunity against human bacterial pathogens. Front. Microbiol..

[26] Liyanage S., Dassanayake R.S., Bouyanfif A., Rajakaruna E., Ramalingam L., Moustaid-Moussa N., Abidi N. (2017). Optimization and validation of cryostat temperature conditions for trans-reflectance mode FTIR microspectroscopic imaging of biological tissues. MethodsX.

[27] Lasch P., Haensch W., Naumann D., Diem M. (2004). Imaging of colorectal adenocarcinoma using FT-IR microspectroscopy and cluster analysis. Biochim. Biophys. Acta Mol. Basis Dis..

[28] Kumar P.N., Swapna T.H., Khan M.Y., Daddam J.R., Hameeda B. (2017). Molecular dynamics and protein interaction studies of lipopeptide (Iturin A) on α- amylase of *Spodoptera litura*. J. Theor. Biol..

[29] Hussein N.N.A., Daddam J.R., Prasad E.M., Naidu N. (2017). Evaluation of novel curcumin derivatives against ethicillin resistant *Staphylococcus aureus* (MRSA). Int. J. Appl. Biol. Pharm..

[30] Suresh B.B.M., Jayasimharayalu L.N. (2017). In silico docking studies of elytraria acaulis gas chromatography-mass spectroscopy derived compound against breast cancer target proteins. World J. Pharm. Pharm. Sci..

[31] Papathoti N., Lingampally N., Parameshwar J., Khan M., Rayalu J., Hameeda B. (2016). In silico and in vitro studies of fungicidal nature of lipopeptide (Iturin A) from *Bacillus amyloliquefaciens* RHNK 22 and its plant growth promoting traits. Indian Phytopathol..

[32] Urama D.T., Tarigopula S., Pasha K., Daddam J. (2016). Homology modelling and structure of Neplanocin A derivative. Onl. J. Bioinform..

[33] Daddam J.R., Rao M., Rao D.S. (2015). Phytochemical screening and anti microbial studies of hemidesmus indicus. Int. J. Appl. Biol. Pharm..

[34] Beda P.D., Veluda S.G., Daddam J.R. (2015). Anti-tubercularl activity and molecular docking of dihydro-pyrimidinone derivatives. Onl. J. Bioinform..

[35] Tsai B.C.-K., Kuo W.-W., Day C.H., Hsieh D.J.-Y., Kuo C.-H., Daddam J., Chen R.-J., Padma V.V., Wang G., Huang C.-Y. (2020). The soybean bioactive peptide VHVV alleviates hypertension-induced renal damage in hypertensive rats via the SIRT1-PGC1α/Nrf2 pathway. J. Funct. Foods.

[36] Smita S., Singh K., Akhoon B., Gupta S., Gupta S. (2013). Bioinformatics tools for interpretation of data used in molecular identification. Analyzing Microbes.

[37] Gupta S.K., Srivastava M., Osmanoglu Ö., Dandekar T. (2020). Genome-wide inference of the *Camponotus floridanus* protein-protein interaction network using homologous mapping and interacting domain profile pairs. Sci. Rep..

[38] Gupta S., Osmanoğlu Ö., Srivastava M., Bencurova E., Dandekar T. (2020). Pathogen and host-pathogen protein interactions provide a key to identify novel drug targets. Reference Module in Biomedical Sciences.

[39] Bencurova E., Gupta S.K., Sarukhanyan E., Dandekar T. (2018). Identification of antifungal targets based on computer modeling. J. Fungi..

[40] Selvaraj C., Singh P., Singh S.K. (2014). Molecular insights on analogs of HIV PR inhibitors toward HTLV-1 PR through QM/MM interactions and molecular dynamics studies: Comparative structure analysis of wild and mutant HTLV-1 PR. J. Mol. Recognit..

[41] Selvaraj C., Panwar U., Dinesh D.C., Boura E., Singh P., Dubey V.K., Singh S.K. (2021). Microsecond MD simulation and multiple-conformation virtual screening to identify potential anti-COVID-19 inhibitors against SARS-CoV-2 main protease. Front. Chem..

[42] Chinnasamy S., Selvaraj G., Selvaraj C., Kaushik A.C., Kaliamurthi S., Khan A., Singh S.K., Wei D.Q. (2020). Combining in silico and in vitro approaches to identification of potent inhibitor against phospholipase A2 (PLA2). Int. J. Biol. Macromol..

[43] Selvaraj C., Selvaraj G., Mohamed Ismail R., Vijayakumar R., Baazeem A., Wei D.-Q., Singh S.K. (2021). Interrogation of *Bacillus anthracis* SrtA active site loop forming open/close lid conformations through extensive MD simulations for understanding binding selectivity of SrtA inhibitors. Saudi J. Biol. Sci..

