# Supplementary figures and images for: Identification of a Chitooligosaccharide Mechanism against Bacterial Leaf Blight on Rice by In Vitro and In Silico Studies
Source: Int J Mol Sci. 2021 Jul 27;22(15):7990. doi: 10.3390/ijms22157990 (PMC8347687; doi:10.3390/ijms22157990)

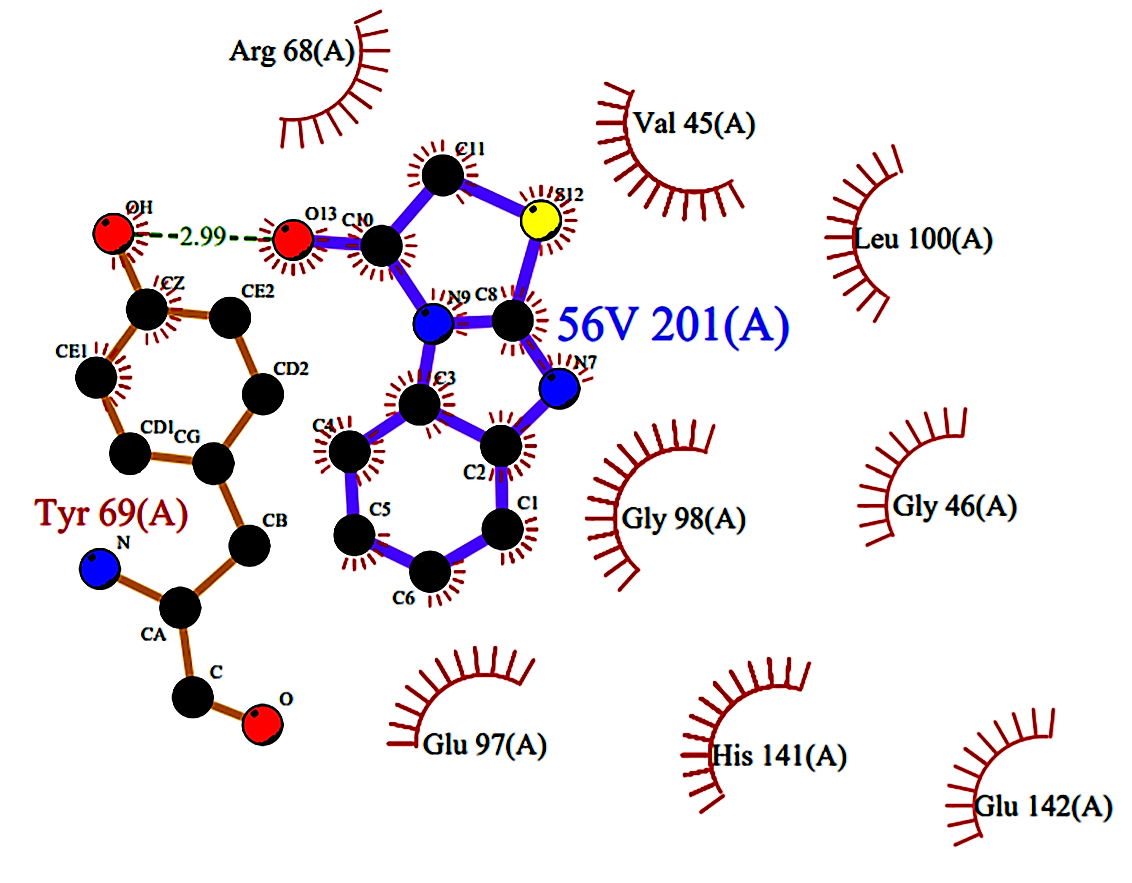

Supplement: Supplementary file 1 [file ijms-22-07990-s001.zip › ijms-1287948-supplementary.tif]
